# Common *HTR2A* variants and 5‐HTTLPR are not associated with human in vivo serotonin 2A receptor levels

**DOI:** 10.1002/hbm.25138

**Published:** 2020-07-22

**Authors:** Marie Spies, Arafat Nasser, Brice Ozenne, Peter S. Jensen, Gitte M. Knudsen, Patrick M. Fisher

**Affiliations:** ^1^ Neurobiology Research Unit, Rigshospitalet Copenhagen Denmark; ^2^ Department of Psychiatry and Psychotherapy Medical University of Vienna Vienna Austria; ^3^ Department of Public Health, Section of Biostatistics University of Copenhagen Copenhagen Denmark

**Keywords:** 5‐HTTLPR, positron emission tomography, serotonin 2A receptor, single nucleotide polymorphism

## Abstract

The serotonin 2A receptor (5‐HT2AR) is implicated in the pathophysiology and treatment of various psychiatric disorders. [^18^F]altanserin and [^11^C]Cimbi‐36 positron emission tomography (PET) allow for high‐resolution imaging of 5‐HT2AR in the living human brain. Cerebral 5‐HT2AR binding is strongly genetically determined, though the impact of specific variants is poorly understood. Candidate gene studies suggest that *HTR2A* single nucleotide polymorphisms including rs6311/rs6313, rs6314, and rs7997012 may influence risk for psychiatric disorders and mediate treatment response. Although known to impact in vitro expression of 5‐HT2AR or other serotonin (5‐HT) proteins, their effect on human in vivo brain 5‐HT2AR binding has as of yet been insufficiently studied. We thus assessed the extent to which these variants and the commonly studied 5‐HTTLPR predict neocortex in vivo 5‐HT2AR binding in healthy adult humans. We used linear regression analyses and likelihood ratio tests in 197 subjects scanned with [^18^F]altanserin or [^11^C]Cimbi‐36 PET. Although we observed genotype group differences in 5‐HT2AR binding of up to ~10%, no genetic variants were statistically significantly predictive of 5‐HT2AR binding in what is the largest human in vivo 5‐HT2AR imaging genetics study to date. Thus, in vitro and post mortem results suggesting effects on 5‐HT2AR expression did not carry over to the in vivo setting. To any extent these variants might affect clinical risk, our findings do not support that 5‐HT2AR binding mediates such effects. Our observations indicate that these individual variants do not significantly contribute to genetic load on human in vivo 5‐HT2AR binding.

## Graphical abstract

We evaluated whether common HTR2A single nucleotide polymorphisms rs6313, rs6314, rs7997012, and the 5‐HTTLPR predict 5‐HT2AR binding, assessed with [18F]altanserin and [11C]Cimbi‐36 positron emission tomography in healthy individuals. This is the largest study to date evaluating genetic predictors of a human in vivo serotonin protein (n = 197). Although relative differences of up to 10% were detected, no statistically significant effect was observed. Our results do not support a significant contribution of these individual variants to genetic load on 5‐HT2AR binding.

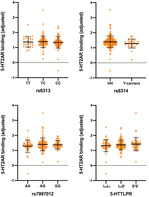

## 1 INTRODUCTION

[^18^F]altanserin and [^11^C]Cimbi‐36 positron emission tomography (PET) provide high‐resolution measures of serotonin 2A receptor (5‐HT2AR) levels in the living human brain (Ettrup et al., 2016; Pinborg et al., 2003). PET studies have shown altered cerebral 5‐HT2AR binding in depression (Mintun et al., 2004), schizophrenia (Rasmussen et al., 2010), and in patients at increased risk for these disorders (Frokjaer et al., 2010; Hurlemann et al., 2005). 5‐HT2AR function mediates antidepressant (Artigas, 2013) and antipsychotic (Meltzer & Massey, 2011) efficacy. A PET study from our group, performed in twins, indicates that cerebral 5‐HT2AR binding is strongly genetically determined (Pinborg et al., 2008). Identifying genetic sources of variation in brain 5‐HT2AR elucidates how variation emerges and highlights mechanisms mediating genetic effects on neuropsychiatric risk.

Candidate gene studies suggest that single nucleotide polymorphisms (SNPs) within the *HTR2A* gene might affect psychiatric risk and treatment effects. rs6311 (i.e., −1438G > A, promotor) and rs6313 (i.e., 102C > T, Exon 1, in perfect linkage disequilibrium) G/C alleles were linked to increased risk for depression (Petit et al., 2014) and schizophrenia (Williams et al., 1996) as well as superior antidepressant (Minov et al., 2001), but lesser antipsychotic response (Arranz et al., 1995). The rs6314 (i.e., 1354C > T, Exon 3) T allele was associated with poor response to antipsychotic treatment (Blasi et al., 2013) and rs7997012 (i.e., IVS2G > A, intronic) A‐carriers may show greater antidepressant response than noncarriers (McMahon et al., 2006). Although recent genome‐wide association studies (GWAS) for depression (Wray et al., 2018) and schizophrenia (Pardinas et al., 2018) do not support significant independent roles in mediating clinical risk, understanding these variants' effects on 5‐HT2AR expression supports elucidation of mechanisms of psychiatric pathophysiology.

In vitro studies are inconclusive regarding these SNP's impact on 5‐HT2AR protein expression. The concordant rs6311/rs6313 A/T alleles showed higher expression in some studies (Parsons, D'Souza, Arranz, Kerwin, & Makoff, 2004; Polesskaya & Sokolov, 2002), though this was contradicted by others (Bray, Buckland, Hall, Owen, & O'Donovan, 2004). rs6314 induces a histidine to tyrosine amino acid change, alters 5‐HT2AR intracellular signaling (Ozaki et al., 1997), and was associated with lower expression levels in vitro and post mortem (Blasi et al., 2013). Effects of rs7997012 on 5‐HT2AR protein levels or function have yet to be reported, though an association with in vivo cerebral serotonin transporter (SERT) binding was observed in humans (Laje et al., 2010). PET provides the unique opportunity to assess whether these SNPs affect 5‐HT2AR binding in the living human brain. The specific impact of individual *HTR2A* SNPs on 5‐HT2AR binding has been insufficiently elucidated. Two previous PET studies failed to demonstrate an effect of the *HTR2A* SNPs assessed here. However, these studies were in small samples (Hurlemann et al., 2008), particularly considering the small effect sizes attributed to singular variants in psychiatry (Bogdan et al., 2017), or only assessed one *HTR2A* variant (Erritzoe et al., 2009).

In addition, a study in marmosets linked a SERT gene (*SLC6A4)* promotor haplotype associated with low SERT expression and an anxiety‐prone phenotype (Santangelo et al., 2016) to reduced brain 5‐HT2AR binding (Santangelo et al., 2019). In humans, 5‐HTTLPR (rs4795541) and the associated rs25531 variant have been associated with depressive pathophysiology (Caspi et al., 2003) and may affect SERT binding (Praschak‐Rieder et al., 2007), though null findings have been observed, including from our lab (Fisher et al., 2017; Murthy et al., 2010). Although associations between 5‐HTTLPR and other serotonin receptors have been detected with PET in humans (Fisher et al., 2012; Lothe et al., 2009), the impact on 5‐HT2AR binding has yet to be investigated.

Here, we aimed to assess whether the aforementioned *HTR2A* SNPs (rs6313, rs6314, rs7997012) and the 5‐HTTLPR predict neocortex 5‐HT2AR binding in the living human brain. To do so, we probed their effects in a uniquely large (*n* = 197) [^18^F]altanserin and [^11^C]Cimbi‐36 PET data set available through the Center for Integrated Molecular Brain Imaging (CIMBI) database (Knudsen et al., 2016). Hereby, we explore whether these variants contribute to genetic load on 5‐HT2AR binding.

## 2 MATERIALS AND METHODS

### 2.1 Participants

All available [^18^F]altanserin and [^11^C]Cimbi‐36 PET data acquired in healthy subjects were extracted from the CIMBI database (Knudsen et al., 2016), providing 291 scans (169/122 [^18^F]altanserin/[^11^C]Cimbi‐36). Eleven scans (two [^18^F]altanserin/nine [^11^C]Cimbi‐36) performed in subjects with self‐reported non‐European ancestry were excluded to limit genetic background confounders and variation in allele frequencies. We restricted our analyses to baseline scans to eliminate pharmacologic intervention effects. Thus, 67 (5/62) rescans as well as eight [^18^F]altanserin scans performed in subjects also scanned with [^11^C]Cimbi‐36 were excluded from analyses. This step resulted in 205 scans (154/51), each performed in a unique individual. Finally, scans without corresponding genotype information were excluded; 5‐HTTLPR data were available in two subjects for whom *HTR2A* variants were not. Thus, our dataset included 197 (154/43) and 195 (153/42) scans, each from a unique individual, for 5‐HTTLPR and *HTR2A* analyses, respectively.

All sampling, including scanning and blood draw for genotyping, took place during participation in studies carried out at the Neurobiology Research Unit, Rigshospitalet, Denmark, that have been detailed previously (Adams et al., 2004; Adams et al., 2005; Erritzoe et al., 2008; Erritzoe et al., 2010; Erritzoe et al., 2011; Ettrup et al., 2014; Ettrup et al., 2016; Frokjaer et al., 2008; Haahr et al., 2015; Hasselbalch et al., 2008; Haugbol et al., 2007; Madsen et al., 2019; Marner et al., 2009; Marner et al., 2011; Marner et al., 2012; Pinborg et al., 2003; Pinborg et al., 2004; Pinborg et al., 2008; Rasmussen et al., 2010). All subjects provided written informed consent and the study protocols were approved by the Ethics Committee of Copenhagen and Frederiksberg, Denmark (KF‐11‐061‐03, KF‐01‐124‐04, KF‐01‐2006‐20, KF‐02‐058‐99, KF‐01‐001‐02, KF‐01‐156‐04, H‐4‐2012‐105, H‐16026898, H‐15004506). Healthy subjects included in the CIMBI database are deemed free from primary psychiatric disorders (according to the DSM‐IV axis 1 or WHO ICD‐10 diagnostic classifications), severe systemic or neurologic disease, pregnancy, head trauma, drug or alcohol abuse, and current or prior intake of psychopharmacologic substances through an interview with a trained clinician. Absence of psychopathologic symptoms is confirmed on the day of the PET‐scan using the symptom check list revised SCL‐90‐R (Derogatis & Savitz, 1999). Additional information on CIMBI database inclusion criteria are detailed in (Knudsen et al., 2016). A largely overlapping dataset was recently utilized by Stenbaek et al., 2019) to assess the impact of openness, a specific personality trait, on 5‐HT2AR binding. Erritzoe et al. (2009) previously assessed one *HTR2A* SNP (rs6311, in perfect LD with rs6313) in a subset (*n* = 95) of the data presented here.

### 2.2 PET imaging

As recently detailed by Stenbaek et al. (2019), [^18^F]altanserin and [^11^C]Cimbi‐36 were synthesized according to Lemaire, Cantineau, Guillaume, Plenevaux, and Christiaens (1991) and Ettrup et al. (2014), respectively. All PET scans were acquired using either an 18‐ring GE‐Advance scanner (General Electric, Milwaukee, WI, *n* = 142 [^18^F]altanserin) or a Siemens ECAT High Resolution Research Tomograph (HRRT, CTI/Siemens, Knoxville, TN, n = 12/43 [^18^F]altanserin/[^11^C]Cimbi‐36). Both were used in 3D acquisition mode and with in‐plane resolutions of approximately 6 mm and <2 mm, respectively (Knudsen et al., 2016). [^18^F]altanserin scans were performed as detailed by Pinborg et al. (2003), using a bolus plus infusion radioligand administration scheme. A 10 min transmission scan was followed by 40 min of dynamic PET scanning (frames: 5 × 8 min), which started 2 hr after initiation of [^18^F]altanserin administration (Pinborg et al., 2003). [^11^C]Cimbi‐36 scans were performed after bolus radioligand administration using a protocol established by Ettrup et al., 2014 and Ettrup et al. (2016). A 6 min transmission scan was followed by 120 min (frames: 6 × 10 s, 6 × 20 s, 6 × 60 s, 8 × 2 min, and 19 × 5 min) of dynamic PET scanning, which commenced with [^11^C]Cimbi‐36 injection. PET data from the GE‐Advance scanner were reconstructed using a filtered back projection algorithm followed by attenuation‐, dead time‐, and scatter correction. Scans performed on the HRRT scanner were reconstructed using a 3D‐OSEM‐PSF algorithm (Hong et al., 2007; Knudsen et al., 2016; Sureau et al., 2008).

### 2.3 MR imaging

Each subject underwent one magnetic resonance imaging (MRI) session for acquisition of a high‐resolution, T1‐weighted structural brain scan. MRI scans were utilized for coregistration, gray matter‐, white matter‐, and cerebrospinal fluid segmentation, as well as region of interest (ROI) delineation. MRI scans were acquired using either a Siemens 1.5 T Vision scanner (Erlangen, DE, *n* = 63 [^18^F]altanserin), a Siemens 3 T Magnetom Trio scanner (*n* = 91 [^18^F]altanserin), a Siemens 3 T Magnetom Verio scanner (*n* = 21 [^11^C]Cimbi‐36) or a Siemens 3 T Prisma scanner (*n* = 22 [^11^C]Cimbi‐36) (Knudsen et al., 2016). See previous publications for scan protocol details (Ettrup et al., 2014; Madsen et al., 2019; Stenbaek et al., 2019).

### 2.4 PET data processing

As previously described (Knudsen et al., 2016; Stenbaek et al., 2019), motion correction utilized the AIR algorithm based on Woods, Cherry, and Mazziotta (1992) and PET scans were smoothed using a 12 or 10 mm within‐frame Gaussian filter for the GE‐Advance and HRRT scanners, respectively. The AIR algorithm was also used for coregistration of scans acquired on the GE‐Advance scanner. For HRRT scans, Statistical Parametric Mapping (SPM8) was used for this purpose.

**FIGURE 1:**
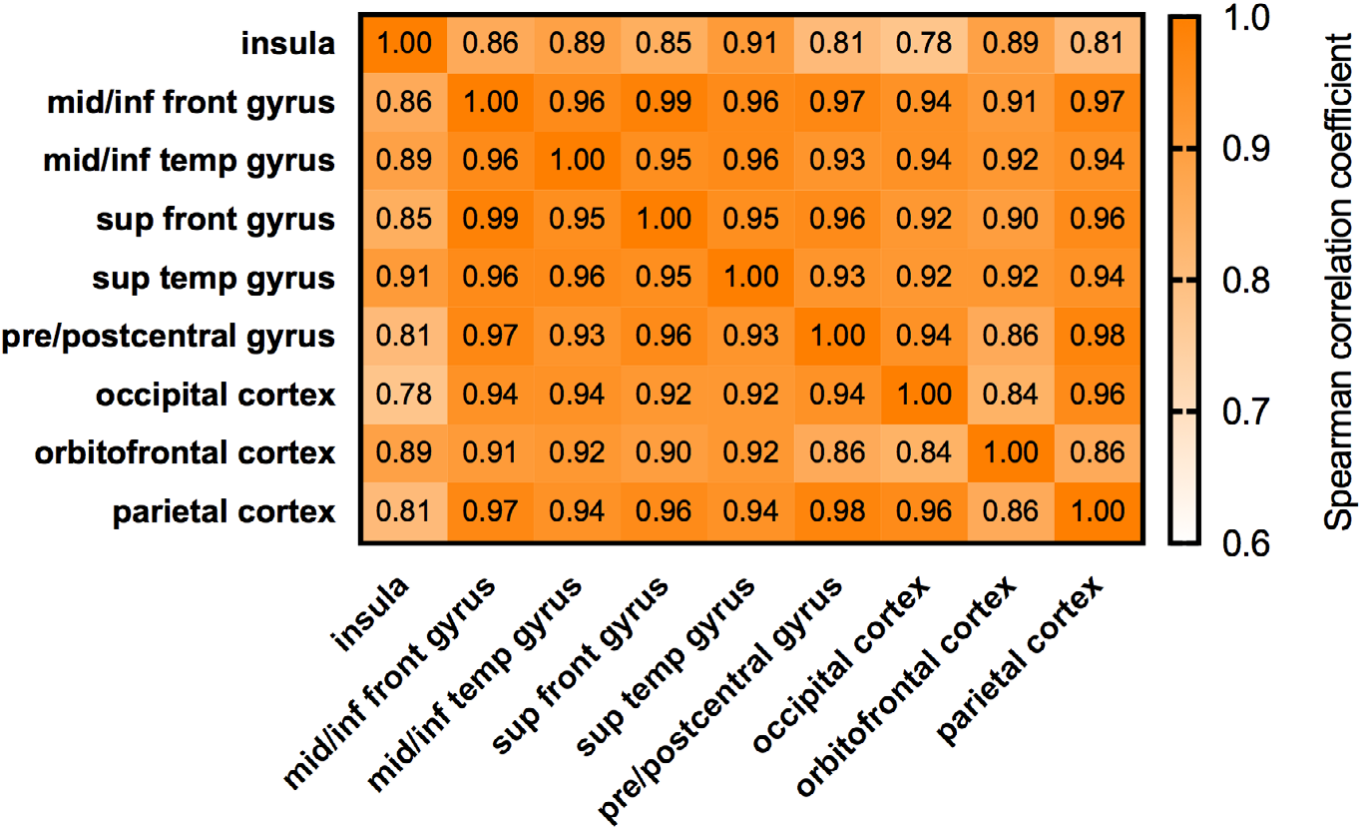
Heat map of correlation coefficients between neocortex ROI subregions. Heat map depicts strength of correlations of 5‐HT2AR binding (pooled [^18^F]altanserin BP_P_: *n* = 154; [^11^C]Cimbi‐36 BP_ND_: *n* = 43) between subregions included in the neocortex ROI and insula. Insula is not included in the PVE lab neocortex ROI, but correlation with neocortex ROI subregions is strong. Each rectangle depicts one region‐to‐region correlation; a darker orange color depicts higher Spearman correlation coefficients. front, frontal; mid/inf, middle/inferior; ROI, region of interest; sup, superior; temp: temporal

[^18^F]altanserin BP_P_ and [^11^C]Cimbi‐36 BP_ND_ were calculated based on Pinborg et al. (2003) and (Ettrup et al. (2014) and Ettrup et al. (2016), respectively. BP_P_ and BP_ND_ are considered indices of 5‐HT2AR density (Innis et al., 2007). The neocortex was defined as the primary ROI and delineated using corresponding subject‐level MRI data and PVElab as published by Svarer et al., 2005). PVElab defines the neocortex to include the middle/inferior frontal‐, middle/inferior temporal‐, superior frontal‐, superior temporal‐, and pre/post central gyri as well as occipital‐, orbitofrontal‐, and parietal cortices. Several biological and methodological characteristics motivated selection of this singular ROI for analyses. Neocortex has high 5‐HT2AR binding, whereas subcortical levels are low (Ettrup et al., 2014) and show lower reproducibility (Ettrup et al., 2016). [^11^C]Cimbi‐36 has serotonin 2C receptor affinity, relevant in hippocampus (Finnema et al., 2014), confounding genetic associations. In the neocortex, BP_P_ and BP_ND_ values from [^18^F]altanserin and [^11^C]Cimbi‐36 scans are correlated (Ettrup et al., 2016), supporting pooling of data. Finally, 5‐HT2AR binding is strongly correlated between neocortical subregions (Figure 1). Thus, our focus on a neocortex ROI represents a statistically efficient strategy for evaluating genetic predictors of brain 5‐HT2AR binding.

### 2.5 Genotyping

Genotyping was done on material from either buffy coat lymphocytes or whole blood samples. Samples or extracted DNA were stored at −20°C in the CIMBI biobank until analyzed. DNA extractions were performed with Qiagen DNA extraction kits (Qiagen, Hilden, Germany, https://www.qiagen.com). Quality control of DNA samples utilized Thermo Scientific NanoDrop spectrophotometry (Thermo Fisher Scientific, Waltham, MA, https://www.thermofisher.com). In case of insufficient purity, DNA decontamination was carried out by sodium acetate precipitation, using 0.1 volume of 3 M sodium acetate and 3 volumes of ice‐cold 96–99% ethanol. To verify consistent genotype results, five previously genotyped rs6313, rs6314, and rs7997012 samples were re‐analyzed with perfect correspondence. rs6311 genotype information was available in 129 subjects and used to confirm perfect LD with rs6313. All other SNPs are in linkage equilibrium based on LDmatrix tool (https://ldlink.nci.nih.gov/?tab=ldmatrix).

All rs6311, rs6313, rs6314, and rs7997012 genotypes were determined using Applied Biosystems TaqMan 5′‐exonuclease allelic discrimination assays (Thermo Fisher Scientific). Previously available genotyping was performed using the ABI 7500 multiplex PCR machine (Thermo Fisher Scientific), while current genotyping utilized the Roche LightCycler 480 II (Roche, Penzeberg, Germany, https://www.lifescience.roche.com).

All 5‐HTTLPR (rs4795541, rs25531) genotyping was performed as described by Madsen et al., 2016). rs4795541 genotyping used TaqMan 5′‐exonuclease allelic discrimination assays and PCR. rs25531 genotyping comprised PCR amplification from the forward and reverse primers 5′‐GGCGTTGCCGCTCTGAATGC‐3′ and 5′‐GAGGGACTGAGCTGGACAACCAC‐3′ followed by MspI restriction enzyme degradation and separation by gel electrophoresis. All kits used in genotyping procedures were utilized according to the manufacturers' instructions.

### 2.6 Statistical analyses

Hardy–Weinberg equilibrium (HWE) was evaluated with a chi‐square test. HWE was not evaluated for 5‐HTTLPR because this variant was an inclusion criterion in some of the original studies.

#### 2.6.1 Genotype grouping

Subjects were grouped for each *HTR2A* variant based on genotype, resulting in three groups for each SNP with the exception of the rs6314, for which we identified only one YY homozygote (i.e., rs6313 TT vs. CT vs. CC; rs6314 HH vs. Y‐carriers; rs7997012 AA vs. AG vs. GG). For the 5‐HTTLPR, L_G_ or S alleles were coded together as an S′ allele and genotypes were grouped accordingly (i.e., S′S′, L_A_S′, L_A_L_A_). This grouping is based on evidence that the L_G_ and S alleles are associated with similarly low SERT expression levels (Hu et al., 2006). See Table 1 for an overview of genotype grouping used in statistical analyses.

**TABLE 1:** Genotype grouping for statistical analyses

Variant	Alleles		
rs6313	TT	CT	CC
	27	88	80
rs6314	HH	Y‐carriers	
	165	30 (one homozygote)	
rs7997012	AA	AG	GG
	45	92	58
5‐HTTLPR^a^	L_A_L_A_	L_A_S′	S′S′
	55	95	47

*Note: HTR2A* variants: *n* = 195. 5‐HTTLPR: *n* = 197.

^a^ 5‐HTTLPR comprises rs4795541 and rs25531 with S′ denoting either composite L_G_ allele or S allele, in accordance with Hu et al. (2006).

#### 2.6.2 Analyses of genotype effects on neocortex 5‐HT2AR binding

[^18^F]altanserin BP_P_ and [^11^C]Cimbi‐36 BP_ND_ data were pooled for analyses and will subsequently be referred to as 5‐HT2AR binding. Continuous variables were mean‐centered prior to model fit. First, the effects of the covariates age, sex, radioligand ([^18^F]altanserin, [^11^C]Cimbi‐36), PET scanner (HRRT, GE), MR scanner field strength (1.5 T, 3 T), and BMI were assessed in a linear regression model. Age, sex (Moses‐Kolko et al., 2011), and BMI (Erritzoe et al., 2009) were previously shown to affect 5‐HT2AR binding. MR scanner field strength, radioligand, and PET scanner were included to address methodological heterogeneity. Interaction effects between radioligand and the covariates age, sex, and BMI were tested for, but were nonsignificant (all *p*_unc_ > .05).

Next, likelihood ratio testing was used to test for a main effect of each variant (rs6313, rs6314, rs7997012, 5‐HTTLPR) by comparing the covariate model with and without each variant. Each variant was evaluated separately. Genotype effects were not moderated by PET ligand (all *p*_unc_ > .05), which is supportive of pooling [^18^F]altanserin BP_P_ and [^11^C]Cimbi‐36 BP_ND_. Likelihood ratio testing was also performed to test for the combined effect of all variants on neocortex 5‐HT2AR. Specific genotype effects were then estimated by linear regression analyses.

Visual and graphical (e.g., QQ plot) evaluation of our model including only covariates indicated nonnormally distributed residuals. This was likely due to inclusion criteria of original studies, some of which specifically recruited overweight (Erritzoe et al., 2009) or elderly individuals (Marner et al., 2009; Marner et al., 2011). To derive estimates robust to this deviation, parameter estimates, 95% confidence intervals (95% CI) and *p*‐values for linear regression models were estimated with 1,000 bootstrap resamples. For all linear regression analyses, only bootstrapped results are reported. Statistical significance of likelihood ratio testing was assessed with a permutation test (10,000 permutations).

#### 2.6.3 Radioligand subgroup models

Linear regression analyses and likelihood ratio testing was also performed within the [^18^F]altanserin (*HTR2A*: *n* = 153; 5‐HTTLPR: *n* = 154) and [^11^C]Cimbi‐36 (*HTR2A*: *n* = 42; 5‐HTTLPR: *n* = 43) subgroups in order to address potential radioligand differences in genotype effects. Analyses were performed as described above for the pooled sample with the exception that covariates lacking more than one level were dropped (i.e., radioligand for both subsamples, PET scanner, and MR field strength for [^11^C]Cimbi‐36).

#### 2.6.4 Exploratory analyses of genotype effects on insula 5‐HT2AR binding

In addition, exploratory analysis probing for effects of 5‐HTTLPR genotype on insula 5‐HT2AR binding was performed in the *n* = 197 pooled sample based on animal studies reporting effects of *SLC6A4* genotype on 5‐HT2AR binding (Santangelo et al., 2019). Again, linear regression analyses and likelihood ratio testing were implemented using the same procedures as for the neocortex ROI.

Statistical analyses were performed using IBM SPSS Software 26.0.0.0 and R (v3.5.0, https://cran.r-project.org/). *p* < .05 was considered statistically significant.

## 3 RESULTS

#### 3.1.1. Descriptive results

See Table 2 for descriptive information. *HTR2A* variants were in HWE (*df* = degrees of freedom, rs6313: *X* ^2^ = 0.13, *df* = 1, *p* = .72; rs6314: *X* ^2^ = 0.05, *df* = 1, *p* = .82; rs7997012: *X* ^2^ = 0.53, *df* = 1, *p* = .47). As expected (Spurlock et al., 1998), rs6311 and rs6313 were in perfect LD, validating the consideration of only rs6313.

**TABLE 2:** Descriptive information

	[^18^F]altanserin (*n* = 154)			[^11^C]Cimbi‐36 (*n* = 43)		
	Mean ± *SD*	Median	Range	Mean ± *SD*	Median	Range
Age (years)	40.76 ± 17.97	35.10	18.47–81.73	26.84 ± 5.81	26.42	18.43–49.82
BMI (kg/m^2^)	25.51 ± 4.70	24.44	18.38–45.91	23.86 ± 2.84	23.08	19.32–33.20
Inj. dose^a^ (MBq)	275.19 ± 64.83	278.00	143.00–447.00	497.74 ± 100.62	511.12	213.00–604.04
SA^a^ (GBq/μmol)	76.14 ± 62.32	55.71	10.30–357.78	345.13 ± 243.01	273.14	73.36–1,039.63
Inj. mass^a^ (μg)	2.84 ± 2.22	2.12	0.26–10.99	0.84 ± 0.48	0.75	0.11–1.50
Neocortical binding^b^	1.46 ± 0.56	1.47	0.32–4.66	1.30 ± 0.21	1.28	0.99–2.02
PET scanner (GE/HRRT)	142/12			0/43		
MR field strength (1.5 T/3 T)	63/91 (1.5 T: Siemens Vision 3 T: Siemens Magnetom Trio)			0/43 (3 T: Siemens Prisma, Siemens Magnetom Verio)		
Sex (m/f)	94/60			25/18		

Abbreviations: BMI, body mass index; HRRT, High Resolution Research Tomograph; Inj., injected (dose/mass); MR, magnetic resonance; PET, Positron emission tomography; SA, specific activity; *SD*, standard deviation.

^a^ Inj. dose: *n* = 129/43; SA: *n* = 81/43; Inj. mass: *n* = 77/43 ([^18^F]altanserin/[^11^C]Cimbi‐36).

^b^ [^18^F]altanserin: BP_P_; [^11^C]Cimbi‐36: BP_ND_.

#### 3.1.2. Covariate model

In the covariate model, age (parameter estimate [95% CI], *p* value: −.02 [−0.02, −0.01], *p* < .001, units: [^11^C]Cimbi‐36 BP_ND_ change per year), radioligand (0.81 [0.31, 1.31], *p* < .001, units: change in 5‐HT2AR binding from [^11^C]Cimbi‐36 BP_ND_ to [^18^F]altanserin BP_P_), and BMI (0.03 [0.002, 0.06], *p* = .04, units: [^11^C]Cimbi‐36 BP_ND_ change per kg/m^2^) had significant effects on neocortex 5‐HT2AR binding. Effects of PET scanner (−0.45 [−0.98, 0.06], *p* = .09, units: [^11^C]Cimbi‐36 BP_ND_ change from HRRT to GE‐Advance scanner), MR field strength (−0.12 [−0.23, 0.001], *p* = .05, units: [^11^C]Cimbi‐36 BP_ND_ change from 3 to 1.5 T) and sex (−0.07 [−0.16, 0.02], *p* = .13, units: [^11^C]Cimbi‐36 BP_ND_ change from female to male) were not statistically significant predictors of 5‐HT2AR binding but nevertheless included when estimating genetic effects.

#### 3.1.3. Genotype effects on neocortex 5‐HT2AR binding

**FIGURE 2:**
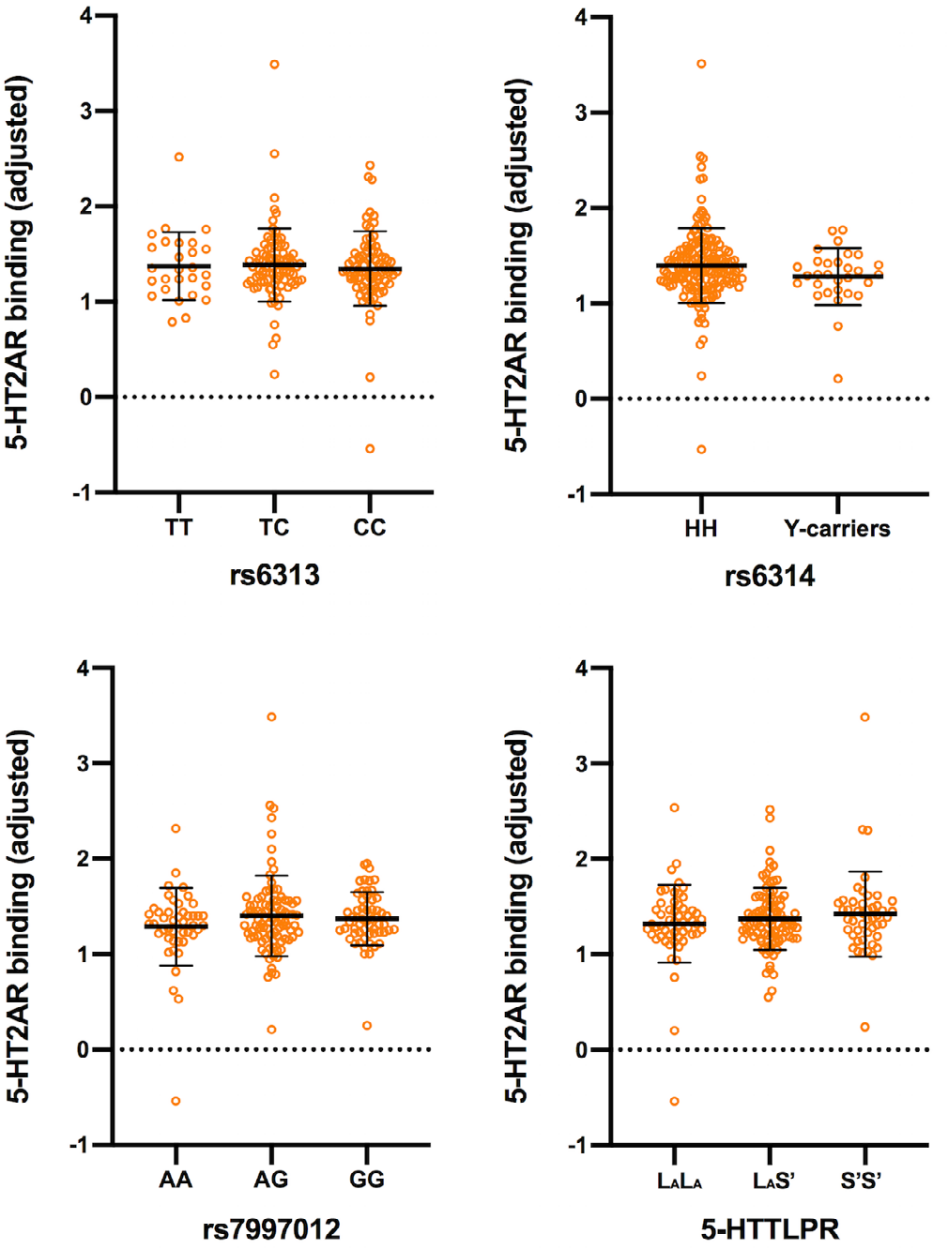
Genotype effects on covariate‐adjusted 5‐HT2AR binding. Orange circles represent covariate‐adjusted (i.e., age, sex, radioligand, positron emission tomography (PET) scanner, MR scanner field strength, and BMI) 5‐HT2AR binding from 195 (*HTR2A* variants) and 197 (5‐HTTLPR) healthy subjects. Negative binding values result from this adjustment, all observed BP values were greater than 0. Black lines illustrate mean ± *SD*. No statistically significant effects of rs6313, rs6314, rs7997012, nor 5‐HTTLPR on covariate‐adjusted 5‐HT2AR binding were observed. See Table 3 for parameter estimates and 95% CI. 5‐HT2AR: serotonin 2A receptor. 95% CI: 95% confidence intervals

We did not observe evidence that any of the genetic variants considered were statistically significant predictors of 5‐HT2AR binding (likelihood ratio estimation effects, rs6313: *X* ^2^ = 0.38, *p* = .84; rs6314: *X* ^2^ = 2.15, *p* = .15; rs7997012: *X* ^2^ = 2.70, *p* = .28; 5‐HTTLPR: *X* ^2^ = 1.80, *p* = .43; all variants combined: *X* ^2^ = 7.56, *p* = .42). See Figure 2 for depiction of specific genotype effects and Table 3 for respective parameter estimates, 95% CI, and % differences in 5‐HT2AR between genotype groups.

**TABLE 3:** Genotype effects on neocortical 5‐HT2AR binding

Genotype/comparison	Estimate^a^	95% CI
rs6313		
TT	1.21	1.06, 1.38
TC	1.22	1.09, 1.36
CC	1.19	1.09, 1.31
TT vs. TC	−0.01 (1.06%)	−0.17, 0.16
TT vs. CC	0.02 (1.42%)	−0.14, 0.18
TC vs. CC	0.03 (2.45%)	−0.09, 0.15
rs6314		
Y‐carriers	1.09	0.93, 1.25
HH	1.21	1.11, 1.32
Y‐carriers vs. HH	−0.11 (10.24%)	−0.24, 0.01
rs7997012		
GG	1.23	1.10, 1.36
AG	1.25	1.13, 1.39
AA	1.14	1.02, 1.27
GG vs. AG	−0.02 (1.97%)	−0.15, 0.10
GG vs. AA	0.09 (7.10%)	−0.06, 0.24
AG vs. AA	0.11 (8.90%)	−0.04, 0.28
5‐HTTLPR^b^		
S′S′	1.24	1.09, 1.40
L_A_S′	1.20	1.10, 1.31
L_A_L_A_	1.15	1.01, 1.28
S′S′ vs. L_A_S′	0.04 (3.34%)	−0.09, 0.18
S′S′ vs. L_A_L_A_	0.09 (7.43%)	−0.07, 0.27
L_A_S′ vs. L_A_L_A_	0.05 (4.23%)	−0.06, 0.17

*Note:* 5‐HT2AR: serotonin 2A receptor. 95% CI: 95% confidence intervals.

^a^ Genotype differences (%) noted where relevant.

^b^ 5‐HTTLPR comprises rs4795541 and rs25531 with S′ denoting either composite L_G_ allele or S allele, in accordance with Hu et al. (2006).

#### 3.1.4. Radioligand subgroup models

We did not observe evidence that any of the genetic variants considered were statistically significant predictors of 5‐HT2AR binding in the [^18^F]altanserin (rs6313: *X* ^2^ = 0.37, *p* = .84; rs6314: *X* ^2^ = 2.56, *p* = .11; rs7997012: *X* ^2^ = 1.32, *p* = .54; 5‐HTTLPR: *X* ^2^ = 1.06, *p* = .61) nor [^11^C]Cimbi‐36 (rs6313: *X* ^2^ = 0.37, *p* = .86; rs6314: *X* ^2^ = .00003, *p* = .99; rs7997012: *X* ^2^ = 5.66, *p* = .08; 5‐HTTLPR: *X* ^2^ = 4.32, *p* = .15) subsamples.

#### 3.1.5. Genotype effects on insula 5‐HT2AR binding

Linear regression analysis and likelihood ratio testing did not reveal an effect of 5‐HTTLPR genotype on insula 5‐HT2AR binding (5‐HTTLPR: *X* ^2^ = 3.00, *p* = .24). All reported results are unadjusted for multiple comparisons.

## 4 DISCUSSION

We did not find evidence for a statistically significant effect of three common *HTR2A* SNPs (rs6313, rs6314, rs7997012), nor the commonly studied 5‐HTTLPR, on 5‐HT2AR binding assessed with [^18^F]altanserin and [^11^C]Cimbi‐36 PET. This represents the largest evaluation of genetic predictors of a human in vivo 5‐HT protein marker to date. Our findings indicate that effects previously shown on gene expression and protein function in vitro (Parsons et al., 2004), post mortem (Blasi et al., 2013; Polesskaya, Aston, & Sokolov, 2006; Polesskaya & Sokolov, 2002; Turecki et al., 1999) and in animal studies (Santangelo et al., 2019) demonstrate only very limited translation to 5‐HT2AR binding in healthy adult humans. Although nominal effects detected for rs6313 and rs6314 were consistent with related in vitro reports (Blasi et al., 2013; Polesskaya & Sokolov, 2002), all observations were not statistically significant. Thus, despite evidence from twin studies for a substantial impact of genetic load on 5‐HT2AR binding (Pinborg et al., 2008), our findings suggest only marginal penetrance of the individual variants considered here. The potential explanations for this disparity differ between the individual SNPs due to their diverging effects on the *HTR2A* gene and how it is regulated.

The rs6311/rs6313 A/T‐alleles have been linked to increased promotor activity (Parsons et al., 2004), mRNA levels (Polesskaya & Sokolov, 2002) and post mortem 5‐HT2AR binding (Turecki et al., 1999). In our sample, TC and TT individuals showed nominally increased 5‐HT2AR binding relative to CC individuals, but observed group differences were ≤2.5% and not statistically significant. Thus, although we did not reject our null hypothesis, we observed effects nominally similar to previous in vitro and post mortem studies (Parsons et al., 2004; Polesskaya & Sokolov, 2002; Turecki et al., 1999). Importantly, rs6311/rs6313 may regulate transcription in a manner moderated by epigenetic mechanisms, including gene methylation (Polesskaya et al., 2006) and interaction with transcription factors (Falkenberg, Gurbaxani, Unger, & Rajeevan, 2011; Smith et al., 2013). Thus, we cannot rule out that epigenetic processes obscured main effects of this variant on 5‐HT2AR binding. Although epigenetic markers can be assayed from peripheral blood samples, this information is not available for the current data and the correspondence between peripheral blood and relevant neuronal populations is unclear. The above mentioned positive post mortem studies suggest that genotype effects override epigenetic‐induced variance. However, negative post mortem studies have also been published (Blasi et al., 2013; Bray et al., 2004). The susceptibility of these variants to epigenetic influence may explain these discrepancies, particularly as the corresponding studies were performed in small samples. In our uniquely large healthy adult sample, we estimate a small, nonsignificant main effect of rs6313 on 5‐HT2AR binding.

Previous studies linked rs6314 HY genotype to lower post mortem prefrontal mRNA levels (Blasi et al., 2013). Although not statistically significant (*p* = .15), we estimated a nominally consistent 10.2% lower 5‐HT2AR binding in Y‐carriers compared to HH homozygotes. rs6314 results in an amino acid change (histidine to tyrosine) (Ozaki et al., 1997). rs6313 is also exonic and could thus potentially also alter protein structure, though it has not been demonstrated in in vitro studies. Such conformational changes could influence binding of [^18^F]altanserin or [^11^C]Cimbi‐36 to 5‐HT2AR. For example, they may influence the affinity of these radioligands for 5‐HT2AR, K_D_, which is inversely proportional to binding potential. To our knowledge, rs6313 or rs6314 effects on radioligand affinity have not been evaluated. However, if these variants were to proportionally affect both available 5‐HT2AR (i.e., B_avail_) and radioligand K_D_, their effects might not be elucidated in our study. Generally, conformational or functional changes to 5‐HT2AR that do not affect [^18^F]altanserin or [^11^C]Cimbi‐36 binding would not be detected with PET. Our binding outcome is thus likely insensitive to rs6314‐induced changes in intracellular signaling (Ozaki et al., 1997). Both [^18^F]altanserin and [^11^C]Cimbi‐36 likely bind intracellularly localized 5‐HT2AR_,_ which comprises ~90% of 5‐HT2AR (Cornea‐Hebert, Riad, Wu, Singh, & Descarries, 1999); it is unknown whether internalization alters [^18^F]altanserin and [^11^C]Cimbi‐36 binding. If unaffected, variant effects on subcellular localization would not be detected in our analyses. In summary, the numerically lower 5‐HT2AR binding observed in the HY genotype is consistent with previous studies (Blasi et al., 2013), yet not statistically significant.

We did not observe a statistically significant impact of rs7997012 on 5‐HT2AR binding. rs7997012 A allele was previously associated with improved antidepressant treatment response (McMahon et al., 2006). Neither in vitro nor in vivo effects of rs7997012 on *HTR2A* gene expression have been reported previously, but an earlier PET study reported a marginal effect on SERT binding (Laje et al., 2010). We estimated 8.9 and 7.1% increases in 5‐HT2AR binding in AG and GG groups compared to AA individuals, respectively; AG and GG individuals showed similar 5‐HT2AR binding (~2% difference). In two cohorts, our lab has reported a nonlinear relation between 5‐HT2AR and SERT binding (Erritzoe et al., 2010; Haahr et al., 2015). Thus, it is plausible that small rs7997012 effects on 5‐HT2AR binding mediate effects on SERT, a model that requires further evaluation in independent samples. in vitro expression studies would support inference of the nominal differences that we observed, resolving rs7997012 functional effects.

We did not observe a statistically significant effect of 5‐HTTLPR on 5‐HT2AR binding. Our data nominally indicate that L_A_S′ and S′S′ status are associated with 4.2 and 7.4% higher 5‐HT2AR binding compared to L_A_L_A_ individuals, a somewhat linear effect. Notably, this remains not statistically significant even when considering L_A_L_A_ versus S′‐carriers (*p* = .27, data not shown), emphasizing a large degree of statistical uncertainty. We performed an exploratory analysis probing for an effect of 5‐HTTLPR on insula 5‐HT2AR binding, which was also nonsignificant (*p* = .24). This was motivated by a recent marmoset study showing an *SLC6A4* haplotype associated with heightened anxiety and reduced insula 5‐HT2AR binding (Santangelo et al., 2016; Santangelo et al., 2019). The convergence with an anxiety phenotype is intriguing considering similar 5‐HTTLPR effects (Lesch et al., 1996). We observed nominally higher 5‐HT2AR with the S allele, as was observed within our neocortex ROI, inconsistent with what would be expected. However, the 5‐HTTLPR polymorphism does not occur in marmosets and is thus not included in the studies by Santangelo et al., who assess a different *SLC6A4* variant. Thus, although we express caution in drawing a direct parallel to (Santangelo et al., 2016), our human in vivo results do not reflect their observations.

The variants included in this study were chosen based on their impact on 5‐HT protein expression (Blasi et al., 2013; Hu et al., 2006; Laje et al., 2010; Polesskaya et al., 2006), allele frequencies, as well as reported associations with disease risk (Caspi et al., 2003; Choi et al., 2004; Petit et al., 2014; Williams et al., 1996) and treatment outcomes of affective and psychotic disorders from candidate gene studies (Anttila et al., 2007; Arranz et al., 1995; Arranz et al., 1998; Blasi et al., 2013; Chen, Shen, & Chen, 2009; Choi, Kang, Ham, Jeong, & Lee, 2005; Kato et al., 2006; McMahon et al., 2006; Minov et al., 2001; Peters et al., 2009). This was done to allow for cautious interpretation of genotype effects vis‐à‐vis clinical findings. 5‐HT2AR binding appears altered in depression (Mintun et al., 2004) and schizophrenia (Rasmussen et al., 2010), and receptor function is modulated by antidepressant and antipsychotic drugs (Artigas, 2013; Meltzer & Massey, 2011). Thus, genetic influence on 5‐HT2AR binding could potentially moderate clinical effects. The relative effects we observed between genotype groups were <10% and not statistically significant, which does not support this assumption. It should be kept in mind that previous clinical effects were observed in diseased populations, whereas we assessed healthy subjects here. It is unclear whether our findings carry over to clinical populations, based on potential genotype by diagnosis interaction effects.

Our study is not without limitations. [^18^F]altanserin and [^11^C]Cimbi‐36 PET data were pooled, introducing data heterogeneity. Radioligand differences in K_D_ as well as differences between [^18^F]altanserin f_P_ and [^11^C]Cimbi‐36 f_ND_ may confound this data pooling, although such confounds are approximated by the inclusion of PET tracer as a covariate. Notably, we observed nonsignificant effects considering each radioligand separately. However, such a split‐sample strategy also negatively affects statistical power. Our healthy population varied in, for example, age and BMI. Although this can introduce measurement error and decrease statistical power, we note that collecting 200 datasets at only two PET scanners is a particularly homogenous sample than is otherwise available. Variation in ancestry was addressed by excluding from analyses persons self‐reported to be of non‐European ancestry. A limitation of the current study is that genetic relatedness and ethnic heterogeneity is not available and not directly modeled. The limitation of evaluating candidate genetic variants as predictors of brain imaging measures has been considered at length (Bogdan et al., 2017). In particular, recent GWAS do not support significant independent roles for the variants assessed here in depression (Wray et al., 2018) or schizophrenia (Pardinas et al., 2018). However, the sample sizes necessary for GWAS are currently not feasible in conjunction with human in vivo PET data. In the absence of available genome‐wide data, focusing on variants selected based on in vitro evidence and clinical candidate gene studies is a potentially useful alternative strategy. In addition, we point out that our outcome measure (5‐HT2AR binding) is more proximal to a genetic effect on protein levels vis‐à‐vis functional and structural brain MRI markers.

In conclusion, this study assessed the effect of three common *HTR2A* SNPs and the 5‐HTTLPR on 5‐HT2AR binding in the largest sample size to date (*n* = 197). Although we do not find evidence for statistically significant differences, we note effects nominally consistent with previous studies linking these variants to 5‐HT2AR levels and the 5‐HT system more broadly. Our study suggests not more than a limited direct effect of these variants on 5‐HT2AR binding in the adult human brain.

## CONFLICT OF INTEREST

Within the last 3 years, M. S. has received travel grants and speaker honoraria from Janssen and Austroplant. All other authors declare no potential conflict of interest.

## Data Availability

The data that support the findings of this study are available on request from the corresponding author and CIMBI database (http://www.cimbi.dk/db). The data are not publicly available due to privacy or ethical restrictions.
